# Analysis of long‐term survival in multiple myeloma after first‐line autologous stem cell transplantation: impact of clinical risk factors and sustained response

**DOI:** 10.1002/cam4.1283

**Published:** 2017-12-28

**Authors:** Nicola Lehners, Natalia Becker, Axel Benner, Maria Pritsch, Martin Löpprich, Elias Karl Mai, Jens Hillengass, Hartmut Goldschmidt, Marc‐Steffen Raab

**Affiliations:** ^1^ Department of Hematology University Hospital Heidelberg Heidelberg Germany; ^2^ Max‐Eder‐Group Experimental Therapies for Hematologic Malignancies German Cancer Research Center (DKFZ) Heidelberg Germany; ^3^ Division of Biostatistics German Cancer Research Center (DKFZ) Heidelberg Germany; ^4^ Institute of Medical Biometry and Informatics University of Heidelberg Heidelberg Germany; ^5^ National Center for Tumor Diseases (NCT) Heidelberg Germany

**Keywords:** Autologous transplantation, maintenance therapy, multiple myeloma, risk factors, survival analysis

## Abstract

The widespread use of high‐dose therapy and autologous stem cell transplantation (ASCT) as well as the introduction of novel agents have significantly improved outcomes in multiple myeloma (MM) enabling long‐term survival. We here analyze factors influencing survival in 865 newly diagnosed MM patients who underwent first‐line ASCT at our center between 1993 and 2014. Relative survival and conditional survival were assessed to further characterize long‐term survivors. Achievement of complete response (CR) post‐ASCT was associated with prolonged progression‐free survival (PFS) in the whole cohort and with significantly superior overall survival (OS) in the subgroup of patients receiving novel agent‐based induction therapy. Landmark analyses performed at 1, 3, and 5 years post‐ASCT revealed that sustainment of any response had a highly significant influence on survival with no significant differences between sustained CR and sustained inferior responses. Furthermore, outcome was independently improved by administration of maintenance therapy. A subset of patients did experience long‐term survival >15 years. However, conditional survival demonstrated a persistent risk of myeloma‐associated death and cumulative relative survival curves did not show development of a clear plateau, even in prognostically advantageous groups. In conclusion, in this large retrospective study, sustained response after first‐line ASCT was found to be a major prognostic factor for OS independent of depth of sustained response. Administration of maintenance therapy further improved outcome, supporting the hypothesis that interventions to prolong responses achieved post‐ASCT may be essential to reach long‐term survival, especially in the setting of persisting residual disease.

## Introduction

The outcomes of patients with multiple myeloma (MM) have greatly improved over recent decades following both the widespread use of high‐dose therapy and autologous stem cell transplantation (ASCT) and, thereafter, the introduction of novel agents.^1^, ^2^, ^3^ Although still considered a largely incurable disease, younger MM patients with low‐risk International Staging System (ISS) scores and no adverse cytogenetic features can now expect to live for 10 years^4^, ^5^ raising the question whether cure might be possible in a subset of patients.^6^ Achievement of complete response (CR) post‐ASCT has been repeatedly shown to be associated with superior prognosis.^7^, ^8^ However, CR patients seem to represent a heterogeneous group with those having persistent minimal residual disease (MRD) at higher risk of early relapse.^9^, ^10^ Patients who progress early after achieving CR do particularly badly, highlighting the importance of efforts to prolong response duration.^6^, ^11^, ^12^, ^13^ Whether interventions to deepen or prolong the duration of response, such as maintenance therapy, contribute to improved overall survival (OS) outside the setting of clinical trials remains an open question. Therefore, more detailed information on clinical characteristics of long‐term survivors as well as the effect of the depth and duration of response is required.

We here provide real‐world data on the outcomes of MM patients treated with upfront ASCT at our center over 22 years as well as a comprehensive analysis of prognostic factors associated with long‐term survival.

## Patients and Methods

Patients with newly diagnosed MM treated at the University Hospital of Heidelberg, Germany, with high‐dose melphalan supported by single or tandem ASCT as part of their first‐line therapy between March 1993 and July 2014 were retrospectively analyzed. Patients who underwent their first ASCT as part of a later line of therapy were not considered for this analysis. Melphalan was administered at a dosage of 200 mg/m² body surface area which was reduced to 100 mg/m² in case of severe renal insufficiency (creatinine clearance < 40 mL/min). Novel agent‐based induction comprised regimens including either thalidomide, lenalidomide or bortezomib. Response assessment was performed at day 100 after ASCT using EBMT criteria.^14^ Additional response assessment according to the IMWG criteria^15^ adapted to include the response category minimal response (MR), was available for the subset of patients who started treatment after 2007. Given the significantly smaller number of IMWG evaluable patients, results according to EBMT response criteria are presented, if not otherwise indicated. A subset of patients received maintenance therapy after ASCT, mostly with interferon or thalidomide, according to the treating physician's discretion.

Progression‐free survival (PFS) and OS were calculated from the day of first ASCT using the Kaplan–Meier method. Patients proceeding to allogeneic transplantation were censored at that time. Prognostic impact of clinical and therapeutic factors on PFS and OS was evaluated on the basis of hazard ratios (HR) with 95% confidence intervals (CI) from multivariate Cox's proportional hazards regression. Maintenance therapy was considered a time‐dependent event potentially following ASCT. Year of the first ASCT was centered at the median. In case of missing variables, “available case analysis” was performed, that is, a case was deleted when missing a variable required for a particular analysis but included for analyses in which all required variables were present. No missing value imputation was performed.

Landmark analyses at 1, 3, and 5 years after first‐line ASCT were performed to evaluate the impact of possible influence factors, in particular of sustained response, on OS of patients alive at these time points by multivariate Cox's proportional hazards regression models. Patients were differentiated into those with sustained CR (combined with near CR (nCR) in the subgroup analysis of IMWG evaluable patients) at the respective time points, sustained inferior responses, that is, very good partial response (VGPR), partial response (PR), MR or stable disease (SD), (sustained non‐CR), those having lost a prior CR or lost a prior inferior response. A sustained response was defined as a response achieved at day 100 after ASCT and absence of relapse/death until the respective landmark. Patients deceased or censored prior to one of these time points were excluded from the respective model.

Furthermore, in order to assess the evolution of prognosis over time, conditional survival CS(*t*|*s*) which expresses the conditional probability of surviving a further *t* years, given that the patient has already survived *s* years, was calculated as the ratio of two Kaplan–Meier estimates $\hat{S}$ with $\hat{\text{CS}}\left(t|s\right)=\frac{\hat{s}\left(s+t\right)}{\hat{s}\left(s\right)}$.^16^ 95% CIs for CS(*t*|*s*) were calculated using a variation in the standard Greenwood formula for the estimation of CIs in unconditional survival.^17^

In order to normalize the observed survival of MM patients *S*_*o*_(*t*) to the expected survival of the general population *S*_*p*_(*t*) adjusting for age, sex, and calendar year, the cumulative relative survival function *r*(*t*) was calculated as $r\left(t\right)=\frac{S_{o}\left(t\right)}{S_{p}\left(t\right)}$ using the R package “periodR” (version 2.0‐9) including survival probabilities extracted from period life tables published by the German Federal Statistical Office.^18^

All statistical tests were two‐sided and *P*‐values <0.05 were considered statistically significant. Calculations were done using the statistical software environment R (version 3.3.2, www.r-project.org) together with the R packages “periodR” (version 2.0‐9), and “survival” (version 2.40‐1). This retrospective study was approved by the University of Heidelberg's Ethics Committee (S‐337/2009).

## Results

### Patients’ characteristics

865 patients with newly diagnosed MM who proceeded to upfront ASCT were included in this analysis. Median age at diagnosis was 56.6 years (range 24–74 years), 509 were male. Novel agent‐based induction therapy was administered to 358 patients, 258 patients underwent tandem ASCT. Following ASCT, 386 patients received maintenance therapy, mainly with interferon *α* or thalidomide. A total of 78 patients proceeded to allogeneic transplantation. Median follow‐up was 7.1 years (range 0.1–21.8 years). Furthermore details on patients’ characteristics are shown in Table 1, details on induction and maintenance regimens are given in suppl. Table S1.

**Table 1:** Patients’ characteristics

	Patient cohort*n* = 865
Median age [range]	56.6 years [24–74 years]
Sex	
Male	509 (58.8%)
Female	356 (41.2%)
MM type	
IgG	486 (58.8%)
IgA	174 (21.1%)
IgD	16 (1.9%)
IgM	1 (0.1%)
Bence‐Jones	150 (18.2%)
double gammopathy (IgG + IgA)	1 (0.1%)
asecretory	36 (4.2%)
*missing*	*1*
ISS stage	
1	249 (45.7%)
2	169 (31.0%)
3	127 (23.3%)
*missing*	*2*
Creatinine	
<2 mg/dL	743 (86.2%)
≥2 mg/dL	119 (13.8%)
*missing*	*3*
Hemoglobin	
<10 g/dL	233 (29.7%)
≥10 g/dL	551 (70.3%)
*missing*	*81*
Thrombocytes	
<150.000/*μ*L	75 (10.4%)
≥150.000/*μ*L	646 (89.6%)
*missing*	*144*
Lactate dehydrogenase	
≤upper limit of normal	547 (82.1%)
>upper limit of normal	119 (17.9%)
*missing*	*199*
Median *β*2‐microglobulin	3.0 mg/L [2.1–4.8]
Median serum albumin	40.8 g/L [35.5–44.6]
Induction therapy	
Novel agent‐based	358 (41.7%)
Conventional	500 (58.3%)
*missing*	*7*
Maintenance therapy	
No maintenance	371 (49.0%)
interferon *α*	265 (35.0%)
thalidomide	84 (11.1%)
other	37 (4.9%)
*missing*	*108*
Single/Tandem ASCT	
Single ASCT	607 (70.2%)
Tandem ASCT	258 (29.8%)
Year of ASCT	
1992–1995	39 (4.5%)
1996–2000	182 (21.0%)
2001–2005	178 (20.6%)
2006–2010	305 (35.3%)
2011–2014	161 (18.6%)
Median time from diagnosis to first ASCT	6.6 months [5.5–8.4]
Response after ASCT (EBMT)	
Complete response	76 (9.4%)
Partial response	652 (80.7%)
Minor response	34 (4.2%)
Stable disease	10 (1.2%)
Progressive disease	36 (4.5%)
Response after ASCT (IMWG)	
Complete response	15 (3.7%)
Near complete response	152 (37.4%)
Very good partial response	89 (21.9%)
Partial response	120 (29.5%)
Minor response	12 (3.0%)
Stable disease	5 (1.2%)
Progressive disease	14 (3.4%)

Laboratory values assessed at time of diagnosis. For serum *β*2‐microglobulin, serum albumin and time from diagnosis to first ASCT median as well as first and third quartiles are given.

Median PFS for the entire patient cohort was 2.0 years, median OS was 6.7 years. Assessed by EBMT response criteria, a CR at day 100 post‐ASCT was achieved by 76 patients (9.4%) who experienced a median PFS of 2.2 years and a median OS of 7.4 years. In comparison, median PFS in patients with PR was 2.2 years, and 1.6 years in patients with MR; median OS was 6.8 years in PR, 5.7 years in MR, and 0.8 years in PD patients (suppl. Fig. S1). A CR prior to ASCT was achieved by 4.2% of patients. In patients with available response assessment according to IMWG criteria, 15 (3.7%) achieved a CR, 167 (41.0%) a CR or nCR; in the subgroup of patients with novel agent‐based induction, CR post‐ASCT was achieved by 4.5%, CR or nCR by 48.3%. No significant differences in outcome between CR and nCR patients were observed (PFS: *P* = 0.90; OS: *P* = 0.64).

### Multivariate risk factor analysis

Multivariate analysis showed that novel agent‐based induction (HR 0.58, *P* < 0.001), administration of maintenance therapy (HR 0.53, *P* < 0.001) and achievement of CR post‐ASCT (HR 0.69, *P* = 0.01) were significantly associated with prolonged PFS. Older age (HR 1.15, *P* = 0.01) and thrombocytopenia <150.000/*μ*L (HR 1.48, *P* = 0.02) at diagnosis were significant risk factors, a negative trend was seen for ISS stage 3 (HR 1.30, *P* = 0.07). Regarding OS, novel agent‐based induction (HR 0.48, *P* < 0.001) and maintenance therapy (HR 0.48, *P* < 0.001) were significantly associated with superior survival, whereas age (HR 1.35, *P* < 0.001) and thrombocytopenia (HR 1.67, *P* = 0.02) were identified as risk factors (Table 2). Achievement of CR prior to ASCT did not exert a significant impact on PFS (HR 0.75, *P* = 0.16) or OS (HR 0.84, *P* = 0.49) in multivariate analysis. Similarly, having received tandem transplant was not significantly associated with prolonged PFS (HR 0.93, *P* = 0.46) or OS (HR 0.80, *P* = 0.10). Subgroup analysis of different modalities of maintenance therapy showed that maintenance therapy with interferon *α* had a pronounced positive impact on PFS (HR 0.47, *P* < 0.001) and OS (HR 0.42, *P* < 0.001), while novel agent based maintenance, largely consisting of thalidomide, failed to reach statistical significance (PFS: *P* = 0.08; OS: HR 0.80, *P* = 0.34), see Figure S2. A further subgroup analysis of patients receiving bortezomib or lenalidomide maintenance was not possible due to the small sample size of patients receiving these therapies.

**Table 2:** Multivariate analysis of possible influence factors on PFS and OS

Factor	PFS		OS	
	HR (95% CI)	*P*‐value	HR (95% CI)	*P*‐value
Age	1.15 (1.04; 1.28)	**0.01**	1.35 (1.17;1.56)	**<0.001**
Year of ASCT	1.01 (0.99; 1.03)	0.24	1.02 (0.99;1.05)	0.21
MM type (reference: IgG)				
IgA	1.06 (0.84; 1.34)	0.61	1.02 (0.77;1.36)	0.89
IgD	0.70 (0.28; 1.72)	0.44	0.93 (0.34;2.55)	0.89
Bence‐Jones	1.05 (0.83; 1.33)	0.67	1.00 (0.74;1.35)	0.99
ISS stage (reference: 1)				
2	1.05 (0.84; 1.30)	0.69	1.04 (0.79;1.35)	0.80
3	1.30 (0.98; 1.73)	0.07	1.34 (0.94;1.91)	0.10
Laboratory values at diagnosis				
Hemoglobin < 10 g/dL	1.09 (0.87; 1.37)	0.48	0.92 (0.69;1.24)	0.60
Thrombocytes < 150.000/*μ*L	1.48 (1.07; 2.04)	**0.02**	1.67 (1.10;2.52)	**0.02**
Creatinine ≥ 2 mg/dL	0.99 (0.72; 1.38)	0.97	1.32 (0.89;1.96)	0.17
LDH > upper limit of normal	1.11 (0.87; 1.43)	0.40	1.28 (0.94;1.74)	0.11
CR after ASCT	0.69 (0.52; 0.93)	**0.01**	0.82 (0.57;1.17)	0.27
Novel agent‐based induction	0.58 (0.45; 0.74)	**<0.001**	0.48 (0.35;0.67)	**<0.001**
Tandem ASCT	0.93 (0.75; 1.14)	0.46	0.80 (0.61;1.04)	0.10
Maintenance therapy	0.53 (0.42; 0.65)	**<0.001**	0.48 (0.37;0.63)	**<0.001**

In the subgroup of patients treated with novel agent‐based induction, achievement of CR/nCR post‐ASCT (IMWG response assessment) was associated with significantly superior PFS (HR 0.44, *P* < 0.001) and OS (HR 0.44, *P* = 0.005) possibly reflecting a qualitatively superior response following the use of novel agents. However, in contrast to the overall patient population, administration of maintenance therapy, in this cohort largely thalidomide, did not appear to confer superior PFS and OS in this subgroup analysis (suppl. Table S2).

### Landmark analysis

**Figure 1:**
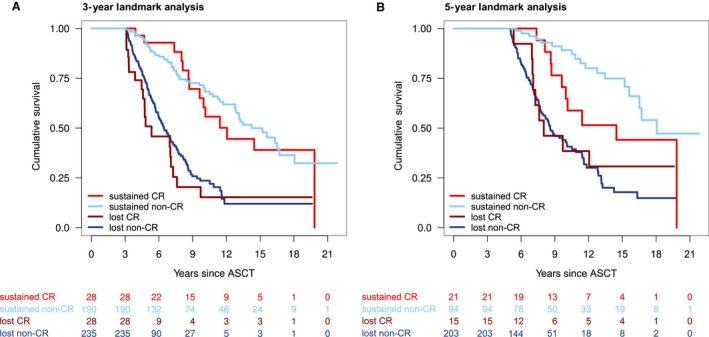
Landmark analyses at 3‐years (A) and 5‐years (B) after ASCT. Patients are stratified by sustained complete response (sustained CR), sustained inferior response (sustained non‐CR), lost CR and lost inferior response (lost non‐CR).

**Figure 2:**
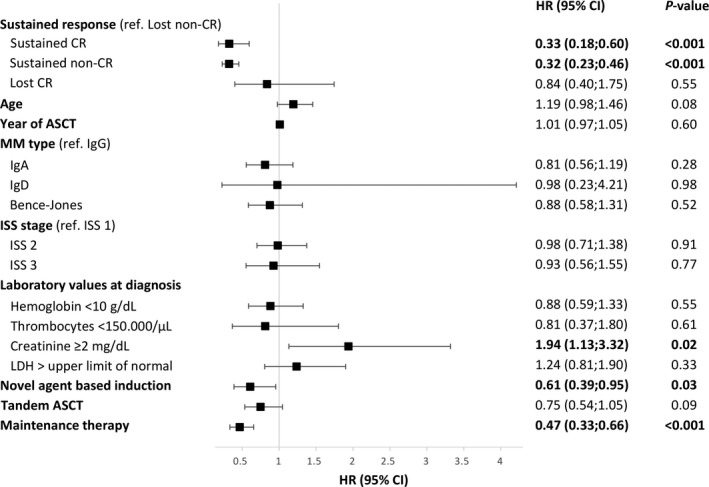
Multivariate analysis of possible influence factors on survival at 3‐year landmark. Hazard ratio (HR), 95% confidence intervals (95% CI) as well as *P*‐values are given.

Landmark analyses were performed at 1, 3, and 5 years post‐ASCT to evaluate the impact of prognostic variables on OS of patients still alive at these time points and, in particular, to assess the effect of response duration. Sustained CR exerted a highly significant positive impact on survival starting at 1, 3, and 5 years after ASCT (HR 0.29, *P* < 0.001, HR 0.33, *P* < 0.001, and HR 0.41, *P* = 0.007, resp.) as did sustained non‐CR (HR 0.35, *P* < 0.001, HR 0.32, *P* < 0.001 and HR 0.21, *P* < 0.001, resp.) (Fig. 1, suppl. Fig. S3). No significant differences were seen between the outcomes of patients with sustained CR compared to those with sustained inferior responses (*P* = 0.37, *P* = 0.98, and *P* = 0.10 for 1, 3, and 5 year landmark analyses, resp.). An independent beneficial effect could be shown at all three landmarks for the administration of maintenance therapy (HR 0.47, *P* < 0.001, HR 0.47, *P* < 0.001 and HR 0.56, *P* = 0.004, resp.) and for novel agent‐based induction (HR 0.44, *P* < 0.001, HR 0.61, *P* = 0.03 and HR 0.50, *P* = 0.03, resp.) (Fig. 2, suppl. Table S3). Assessing the impact of different regimens of maintenance therapy, maintenance therapy with interferon *α* continued to show a pronounced benefit on survival compared to no maintenance (HR 0.42, *P* < 0.001, HR 0.40, *P* < 0.001 and HR 0.48, *P* = 0.001, resp.), while no significant impact could be found for maintenance therapy with novel agents, in our cohort largely thalidomide (HR 0.76, *P* = 0.29, HR 0.94, *P* = 0.83 and HR 1.28, *P* = 0.53, resp.). When analysis was restricted to the subset of patients with novel agent‐based induction (IMWG response assessment), sustained non‐CR/nCR seemed to be inferior to sustained CR/nCR at a 1‐year landmark analysis (HR 2.26, *P* = 0.01), although caution is advisable due to the small number of events in these subgroup analyses.

Landmark analyses thus revealed that a sustained response of any kind appeared to confer a major beneficial effect on survival which was further independently improved by the administration of maintenance therapy.

### Conditional survival

**Figure 3:**
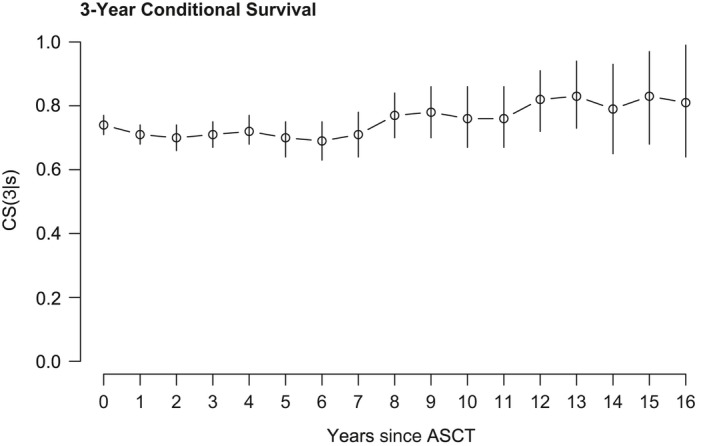
3‐year conditional survival for the entire patient cohort.

We then assessed whether it was possible to determine a minimal survival time which predicted subsequent long‐term survival. We therefore calculated the conditional survival CS(*t*|*s*) as the probability of surviving a further *t* years after having already survived *s* years following ASCT. On analysis of the entire cohort at the time of ASCT (*s *=* *0), 3‐year conditional survival CS(3|*s* = 0) was 74% [95% CI 71%; 77%] and 5‐year conditional survival CS(5|*s* = 0) was 59% [56%; 63%]. While there seemed to be a slight trend towards improved conditional survival over time, no specific minimal survival time of prognostic value for long‐term survival could be defined (Fig. 3, suppl. Fig. S4A). Regarding conditional survival of specific response groups, no apparent differences could be found between patients with CR or PR after ASCT with CS(3|*s* = 0) being 82% [73%; 91%] for CR and 77% [74%; 81%] for PR patients. In contrast, patients with PD at day +100 post‐ASCT had a much lower probability of surviving the following 3 years after ASCT compared to responding patients with a CS(3|*s* = 0) of only 25% [8%; 42%]. At 1 year post‐ASCT, however, the conditional survival *CS*(3|*s* = 1) of the subgroup of patients with PD at day +100 post‐ASCT but still alive 1 year post‐ASCT increased to 58% [27%; 90%] and was thus similar to conditional survival of patients with initial PR (*CS*(3|*s* = 1) = 72% [68%; 76%]) or CR (*CS*(3|*s* = 1) = 72% [61%; 83%]) at that time point (suppl. Fig. S4B).

In summary, assessment of conditional survival revealed that, in our patient cohort, the likelihood of ongoing survival remained relatively stable over time with no evidence of a significantly improved prognosis after a certain time point, once again highlighting the importance of response duration.

### Relative survival

**Figure 4:**
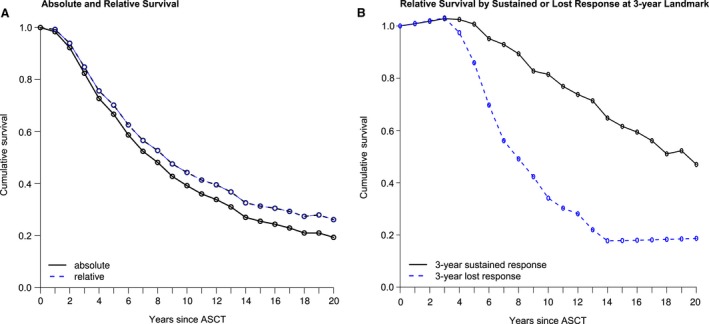
Absolute and relative survival for the entire patient cohort (A) as well as relative survival stratified by sustained response (at 3‐year landmark) (B).

In addition, the relative survival of MM patients was calculated by normalizing against the expected mortality rate in the corresponding general age‐ and sex‐matched population. Relative survival was assessed for the entire MM patient cohort as well as for the subgroups of patients with 3‐year sustained response, novel agent‐based induction therapy, and CR after ASCT (Fig. 4, suppl. Fig. S5). However, no clear plateau suggestive of cure was seen, neither in the overall patient population nor in any of the prognostic subgroups.

## Discussion

Improvements in response rates and overall outcomes of MM patients over recent decades have prompted interest in the more detailed characterization of long‐term survivors and have raised the question of potential cure.^6^, ^8^, ^19^ Modern diagnostic techniques allow for further differentiation of patients with CR into those achieving stringent CR or even MRD negativity, both associated with excellent outcomes.^20^, ^21^ In our cohort, achievement of CR post‐ASCT was associated with prolonged PFS but failed to reach statistical significance with regard to OS. Similar observations were made by several groups in the era prior novel agents,^22^, ^23^, ^24^ possibly indicating that the response obtained by conventional chemotherapeutic agents, though fulfilling the criteria for CR, was of insufficient depth to affect OS. This hypothesis is lent further support by our subgroup analysis of patients who received novel agent‐based induction which found that achievement of CR/nCR post‐ASCT did, in fact, confer significant improvements in both PFS and OS. Consistently, a recent meta‐analysis of 3 first‐line MM trials did not find a superior survival of patients with CR compared to PR in the setting of persistent MRD.^25^

### Residual disease and sustained response

Along these lines, detection of persistent MRD has been identified as a risk factor for early relapse from CR.^9^, ^10^ Loss of CR is associated with adverse outcome, especially if occurring within the first 12–24 months post‐ASCT.^9^, ^13^ Patients with high‐risk cytogenetic features are more likely to relapse early despite promising response rates.^9^, ^26^ In fact, a rapid initial response and rapid subsequent relapse have been observed as features of aggressive disease characterized by a high proliferative index.^27^, ^28^ In contrast, it has been suggested that some MM patients with a presumably MGUS‐like biology experience excellent survival despite failing to achieve CR.^29^, ^30^

It therefore appears that sustainment of response might be at least as important as depth of response.^11^, ^12^ In patients treated with the highly aggressive total therapy regimens, sustained CR was associated with excellent survival, whereas patients relapsing from CR experienced worse outcomes than those never achieving CR.^6^, ^11^, ^12^ In our cohort of patients treated with first‐line ASCT but who received heterogeneous induction and maintenance regimens, sustained response was likewise revealed as a major prognostic factor. The prognostic impact of sustained response remained highly statistically significant in 1, 3, and 5 year landmark analyses suggesting a continued effect. It is worth noting that patients with sustained partial responses also experienced excellent outcomes and in the overall cohort, no significant differences in survival were discernible between patients with sustained CR and sustained inferior responses. In the subgroup of patients with novel agent‐based induction, however, there seemed to be a superior outcome in patients with sustained CR/nCR compared to sustained non‐CR/nCR. This differential effect seen in novel agent treated patients compared to our overall patient cohort might reflect the greater depth of tumor eradication achieved by novel agent‐induced CR compared to CR following conventional chemotherapy.^31^

### Impact of maintenance therapy

In our analysis, administration of maintenance therapy was found to be of major prognostic significance in multivariate cox analysis, multistate models and landmark analyses. Maintenance therapy has been linked to prolongation of PFS with some studies showing an additional OS benefit.^32^, ^33^ The importance of PFS prolongation could be further highlighted in a multistate model of our patient cohort showing time to relapse to be positively associated with post‐relapse survival (data not shown). It is worth noting that our landmark analyses found sustained response and maintenance therapy to be of independent prognostic significance.

When maintenance therapy with interferon *α* and novel agent‐based maintenance (largely thalidomide) were assessed separately, the latter failed to show a significant impact on survival in our patient cohort while maintenance with interferon *α* continued to be a highly significant influence factor on PFS and OS. In clinical trials, thalidomide maintenance did not consistently improve OS^34^, ^35^ and, indeed, might even be harmful in the setting of high‐risk disease.^36^ Regarding maintenance therapy with interferon *α*, two large meta‐analyses of randomized trials showed a significant benefit in terms of time to progression and OS for patients in the interferon *α* trial arms.^37^, ^38^ However, given its toxicity profile as well as the availability of modern agents, its use in MM maintenance therapy has been largely abandoned.^39^ While this retrospective analysis is not powered to evaluate different maintenance regimens, the overall impact of maintenance therapy is certainly impressive and highlights the potential of this treatment modality in improving MM survival.

### Potential cure of MM

Whether MM might ultimately be cured in a significant number of patients remains a matter of debate. Some authors have reported achievement of a plateau in survival curves suggestive of cure in a subset of patients, especially following intense treatment protocols.^6^, ^8^ While some patients in our cohort remained in remission for >15 years following ASCT, they were too few to allow for conservative determination of a clear plateau. It is interesting to note, however, that these long‐term survivors included both patients in CR and PR. Assessment of conditional survival showed a trend toward improving prognosis over time, however, no minimal survival time of prognostic value for long‐term survival could be identified indicating a persisting risk of MM associated death even more than 15 years following ASCT.

Several factors might account for the lack of a demonstrably cured cohort. As our study population spans more than two decades, only 41.7% of patients received novel agent‐based induction therapy, here identified to be a major prognostic factor. Furthermore, as widespread use of novel agents was implemented at our institution starting in 2008, follow‐up time of patients treated with novel agents might be, as yet, inadequate to allow for a clear identification of such a cured cohort.

This is one of the largest analyses of outcomes and prognostic factors in transplant‐eligible MM patients not included in clinical trials. Given the “real world” origin of this data, this analysis is subject to a number of limitations. Our patient cohort is more heterogeneous with respect to both clinical characteristics and treatment approaches than would be found in a clinical trial setting. Certain treatment options, such as a tandem transplant or maintenance therapy, were not administered in a randomized manner but were dependent on the treating physician's discretion. This reflects the evolution of therapeutic strategies and changing availability of novel agents over time. In addition, the CR rate observed in our patient cohort might be underestimated as some patients possibly opted against a bone marrow aspirate required to confirm CR in an out‐of‐trial setting. To compensate for this, we addressed patients with CR and nCR together in the IMWG evaluable cohort. Furthermore, the datasets of certain variables are incomplete. In particular, cytogenetic data was not available in enough patients to be included in this analysis. On the other hand, our data has the important advantage of being more representative of the general MM patient population as the eligibility criteria employed in clinical trials tend to result in a younger fitter cohort than would be observed in routine clinical approach.

In conclusion, in this large retrospective study, we found sustained response after first‐line ASCT to be a strong prognostic factor for OS, not only for those in CR but also for patients with lesser responses. Administration of maintenance therapy further improved outcomes, supporting the hypothesis that interventions prolonging responses achieved post‐ASCT are essential to reach long‐term survival. This needs to be further investigated in current MRD‐driven approaches to determine the roles of duration or depth of response, respectively, in contributing to the long‐term achievement of functional cure of MM patients.

## Conflict of Interest

The authors declare no competing interests.

## Supporting information


**Figure S1.** Progression‐free survival (A) and overall survival (B) stratified by response achieved after ASCT. EBMT response criteria are applied with CR, complete response; PR, partial response, MR, minimal response, and PD, progressive disease. Due to the very small number of patients with stable disease, data not shown.

**Figure S2.** Simon‐Makuch plots of progression‐free survival (A) and overall survival (B) stratified by type of maintenance therapy. Simon‐Makuch plots show PFS and OS according to no maintenance therapy, maintenance therapy with interferon *α* or with novel agents (i.e., thalidomide, bortezomib or lenalidomide). Maintenance therapy is assessed as a time‐dependent variable thus accounting for an individual's possible change from “no maintenance” to “maintenance” over time.

**Figure S3.** Landmark analysis at 1‐year after ASCT. Patients are stratified by sustained complete response (sustained CR), sustained inferior response (sustained non‐CR), loss of complete response (lost CR) and loss of inferior response (lost non‐CR).

**Figure S4.** 5‐year (A) conditional survival for the entire patient cohort as well as 3‐year conditional survival stratified by response achieved after ASCT (B). EBMT response criteria are applied with CR, complete response; PR, partial response; MR, minimal response; SD, stable disease, and PD, progressive disease.

**Figure S5.** Relative survival stratified by type of induction therapy (A) and response achieved after ASCT (B).

**Table S1.** Details on induction regimens. Details on the most commonly applied induction regimens in our cohort are given.**Table S2.** Multivariate analysis of possible influence factors on PFS and OS – subgroup analysis of patients with novel agent‐based induction therapy.**Table S3.** Landmark analyses. Multivariate analysis of possible impact factors on OS is given at 1, 3, and 5 years after ASCT landmarks.
