# Supplementary material for: Analysis of long‐term survival in multiple myeloma after first‐line autologous stem cell transplantation: impact of clinical risk factors and sustained response
Source: Cancer Med. 2017 Dec 28;7(2):307–16. doi: 10.1002/cam4.1283 (PMC5806105; doi:10.1002/cam4.1283)
Supplement: Supplementary file 1 — Figure S1. Progression‐free survival (A) and overall survival (B) stratified by response achieved after ASCT. EBMT response criteria are applied with CR, complete response; PR, partial response, MR, minimal response, and PD, progressive disease. Due to the very small number of patients with stable disease, data not shown. [file CAM4-7-307-s001.pdf]

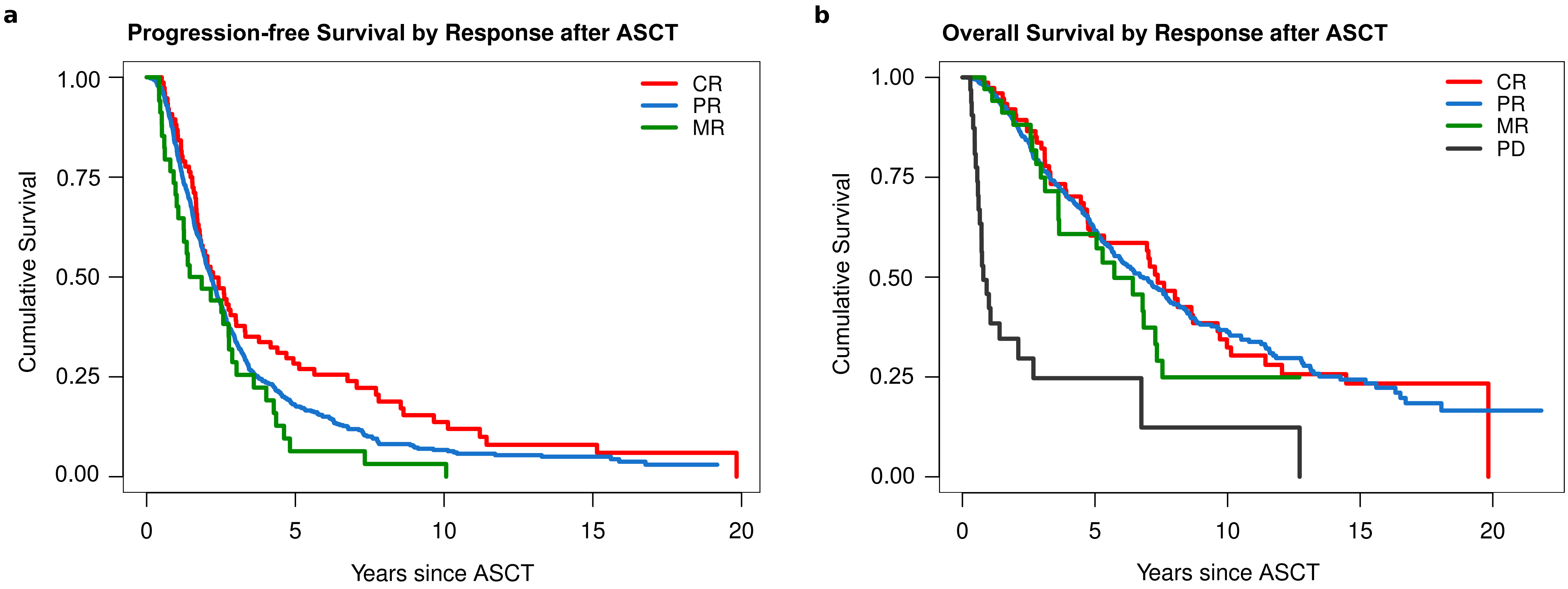

CR	76	42	25	17	11	8	4	4	2	1	1
PR	652	331	129	66	29	22	15	12	7	4	0
MR	34	16	7	2	1	1	0	0	0	0	0

CR	76	65	45	30	19	13	11	7	4	1	0
PR	652	511	315	175	101	62	42	25	10	3	1
MR	34	29	17	11	6	1	0	0	0	0	0
PD	36	6	3	2	1	1	0	0	0	0	0
