# Supplementary figures and images for: Analysis of long‐term survival in multiple myeloma after first‐line autologous stem cell transplantation: impact of clinical risk factors and sustained response
Source: Cancer Med. 2017 Dec 28;7(2):307–16. doi: 10.1002/cam4.1283 (PMC5806105; doi:10.1002/cam4.1283)

**a****Progression-free survival**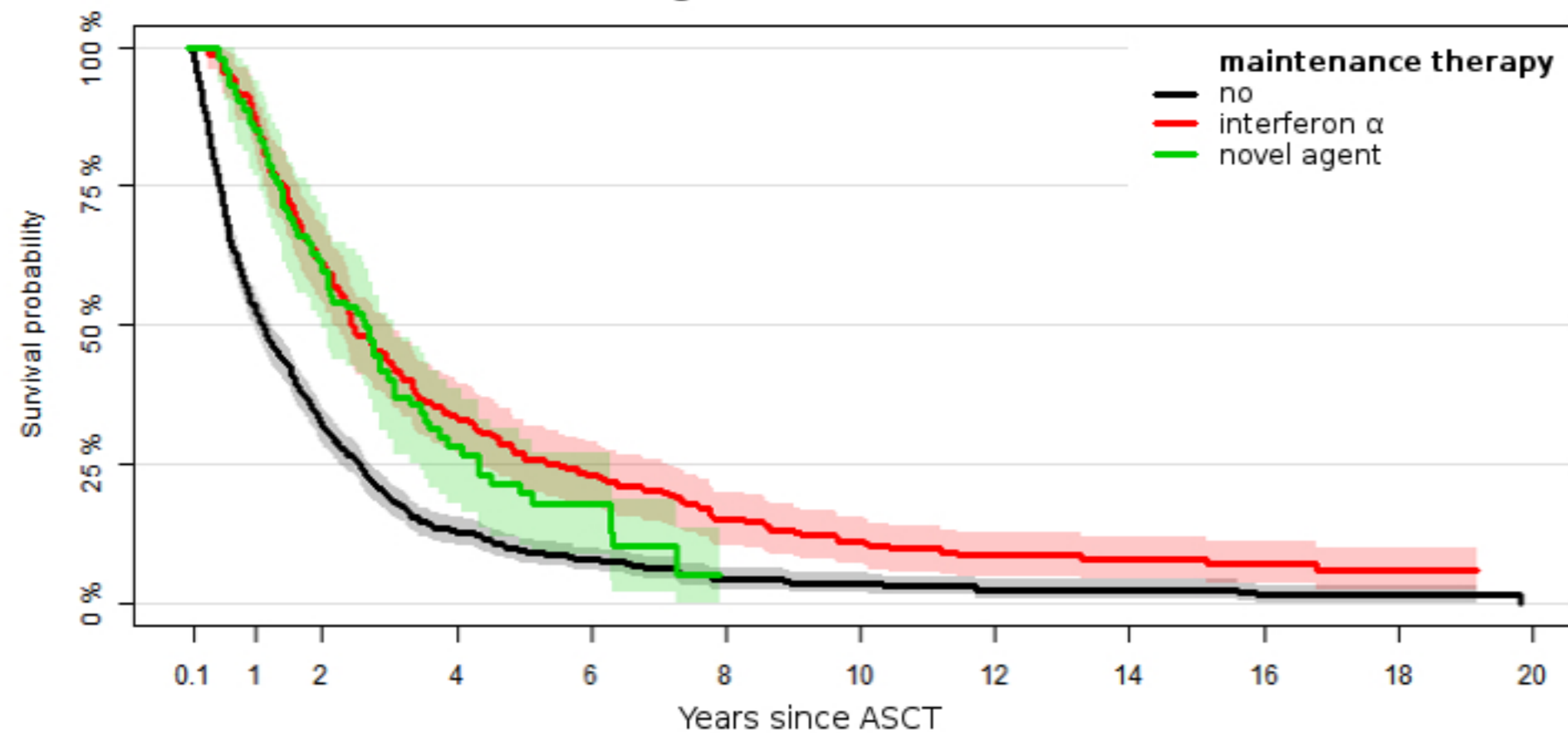**b****Overall survival**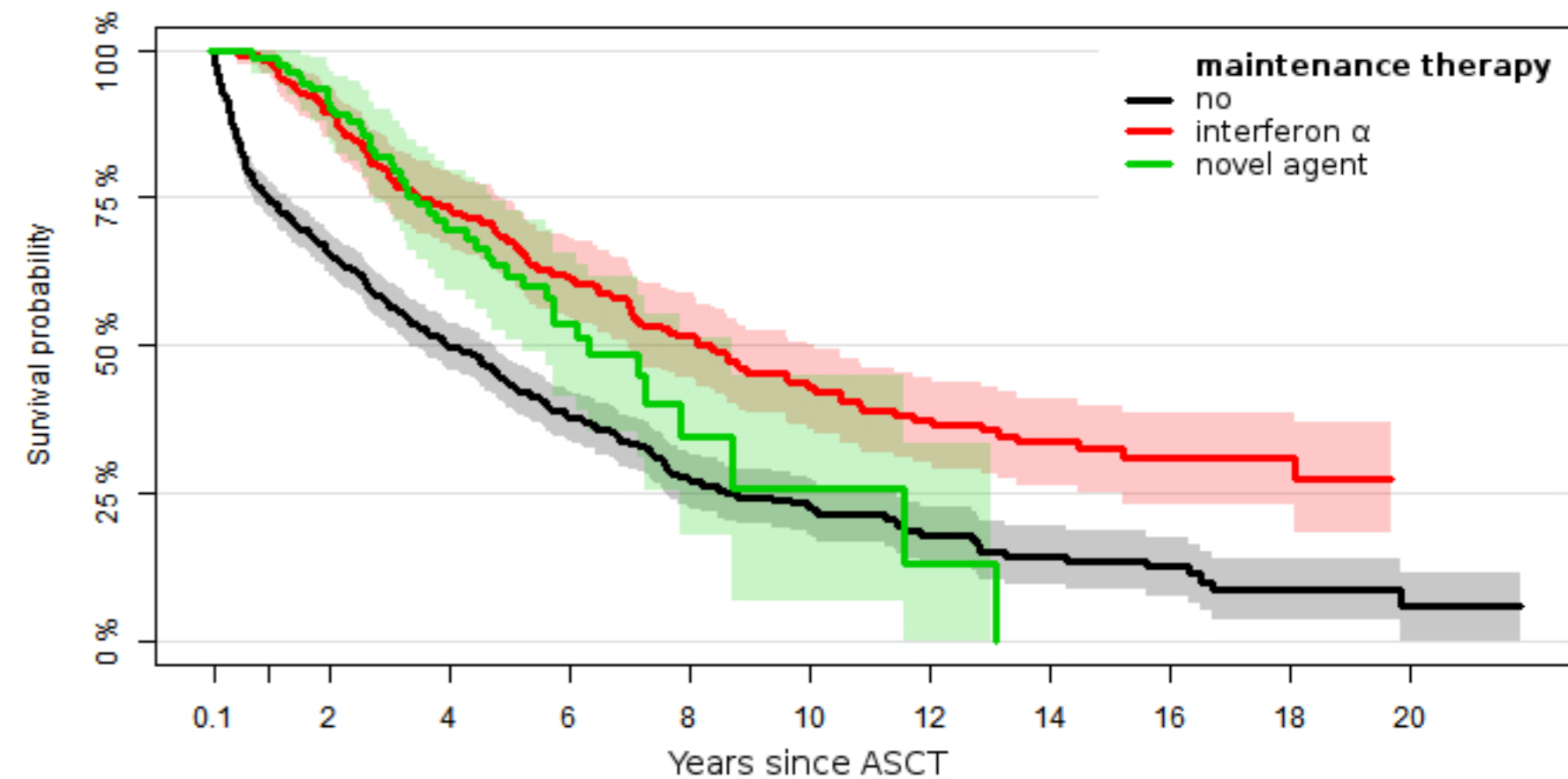

Supplement: Supplementary file 2 — Figure S2. Simon‐Makuch plots of progression‐free survival (A) and overall survival (B) stratified by type of maintenance therapy. Simon‐Makuch plots show PFS and OS according to no maintenance therapy, maintenance therapy with interferon α or with novel agents (i.e., thalidomide, bortezomib or lenalidomide). Maintenance therapy is assessed as a time‐dependent variable thus accounting for an individual's possible change from “no maintenance” to “maintenance” over time. [file CAM4-7-307-s002.pdf]

1-Year Landmark Analysis

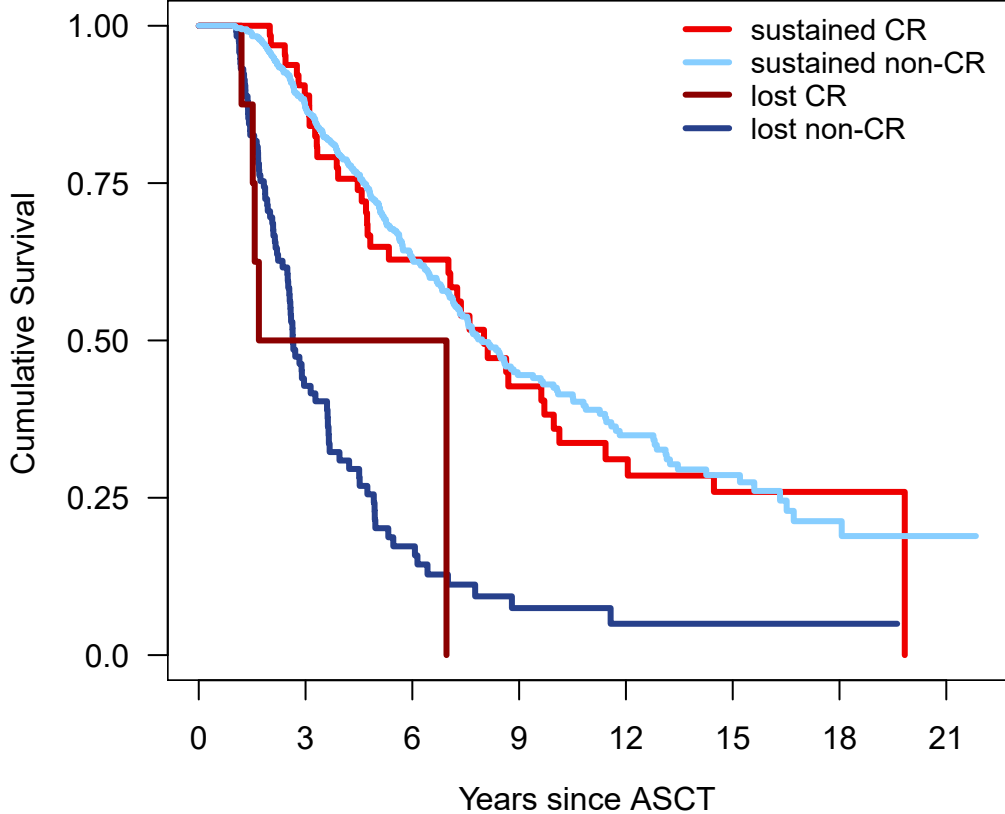

|                  |     |     |     |    |    |    |   |   |
|------------------|-----|-----|-----|----|----|----|---|---|
| sustained CR     | 65  | 55  | 30  | 19 | 12 | 8  | 2 | 0 |
| sustained non-CR | 544 | 390 | 210 | 97 | 50 | 26 | 9 | 1 |
| lost CR          | 8   | 1   | 1   | 0  | 0  | 0  | 0 | 0 |
| lost non-CR      | 118 | 36  | 12  | 4  | 1  | 1  | 1 | 0 |

Supplement: Supplementary file 3 — Figure S3. Landmark analysis at 1‐year after ASCT. Patients are stratified by sustained complete response (sustained CR), sustained inferior response (sustained non‐CR), loss of complete response (lost CR) and loss of inferior response (lost non‐CR). [file CAM4-7-307-s003.pdf]

**a**

### 5-Year Conditional Survival

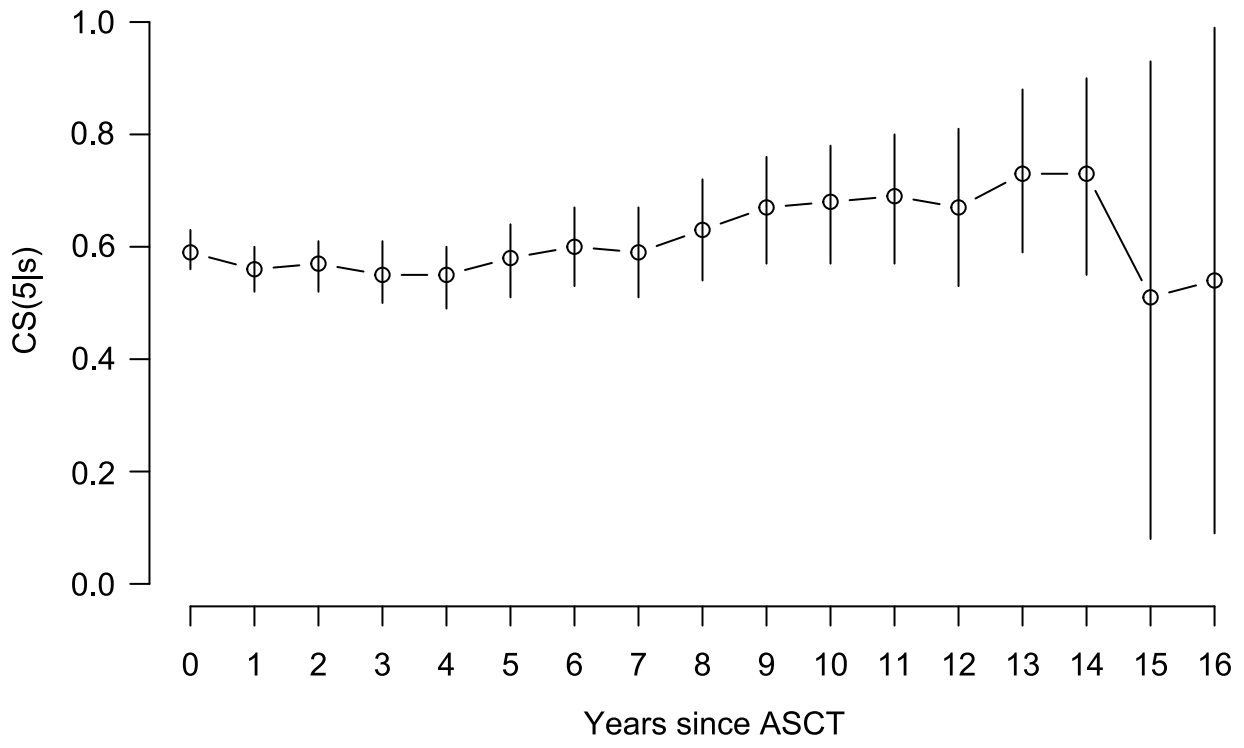**b**

### 3-Year Conditional Survival by Response after ASCT

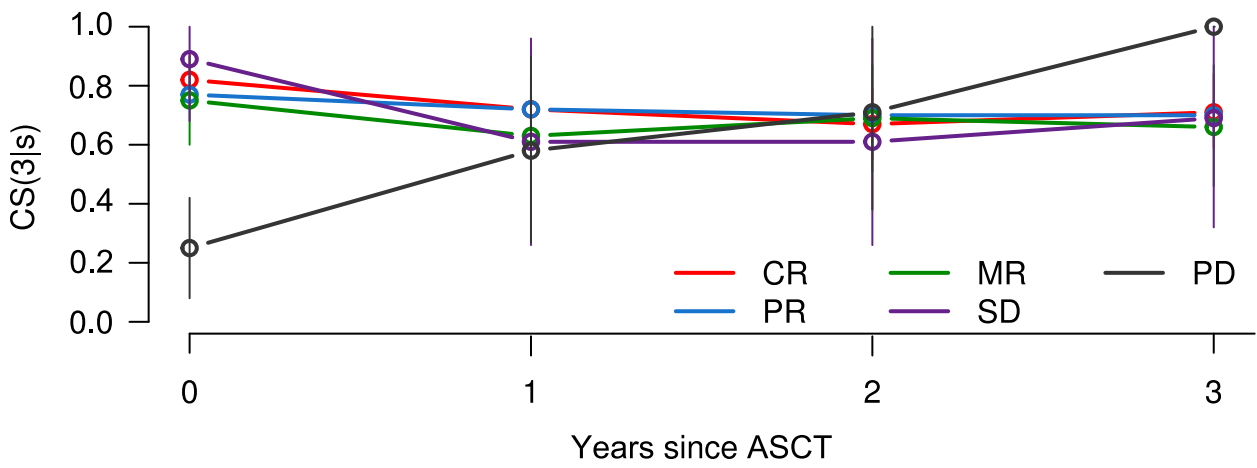

Supplement: Supplementary file 4 — Figure S4. 5‐year (A) conditional survival for the entire patient cohort as well as 3‐year conditional survival stratified by response achieved after ASCT (B). EBMT response criteria are applied with CR, complete response; PR, partial response; MR, minimal response; SD, stable disease, and PD, progressive disease. [file CAM4-7-307-s004.pdf]

**a****Relative Survival by Type of Induction Therapy**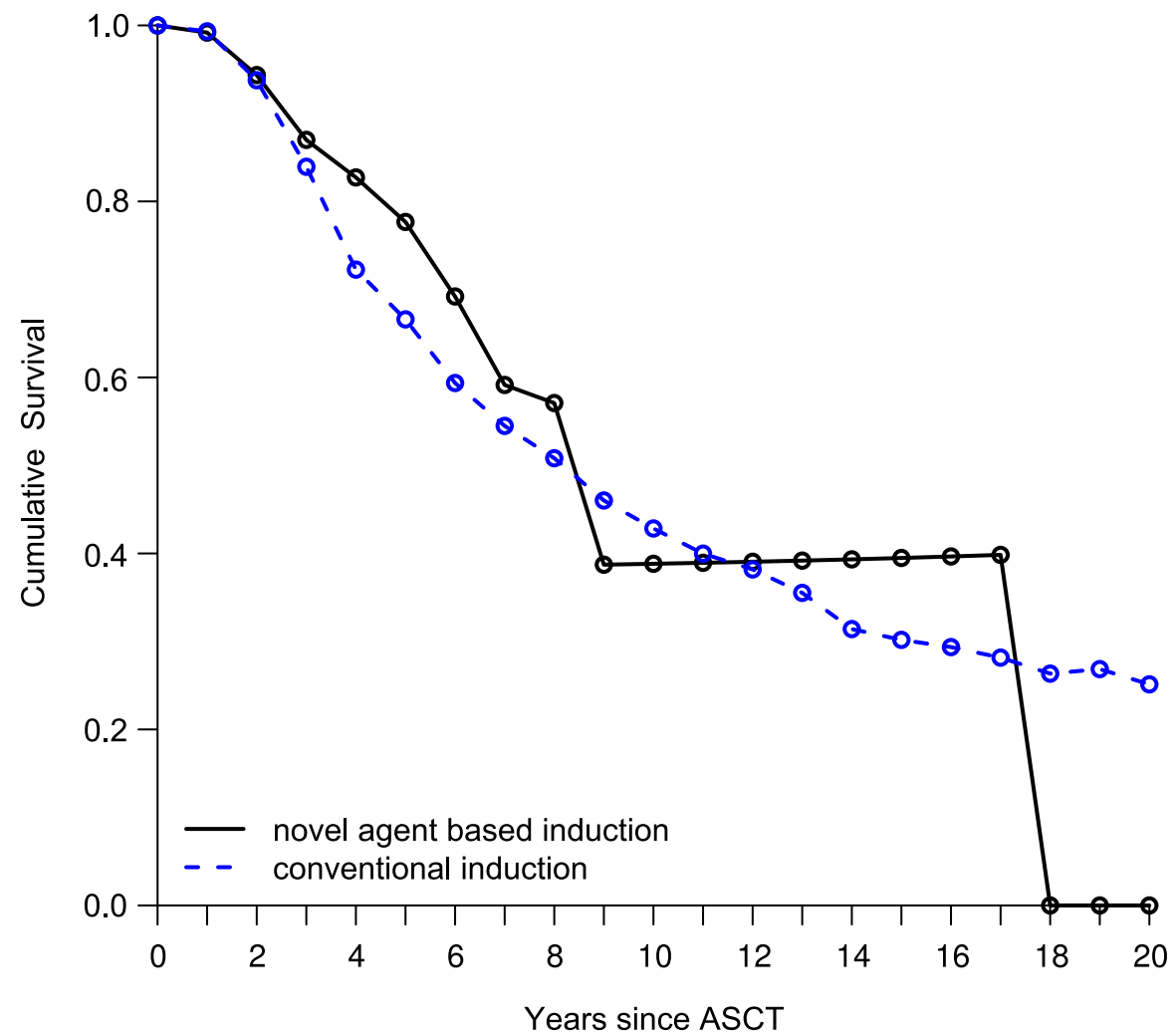**b****Relative Survival by Response after ASCT**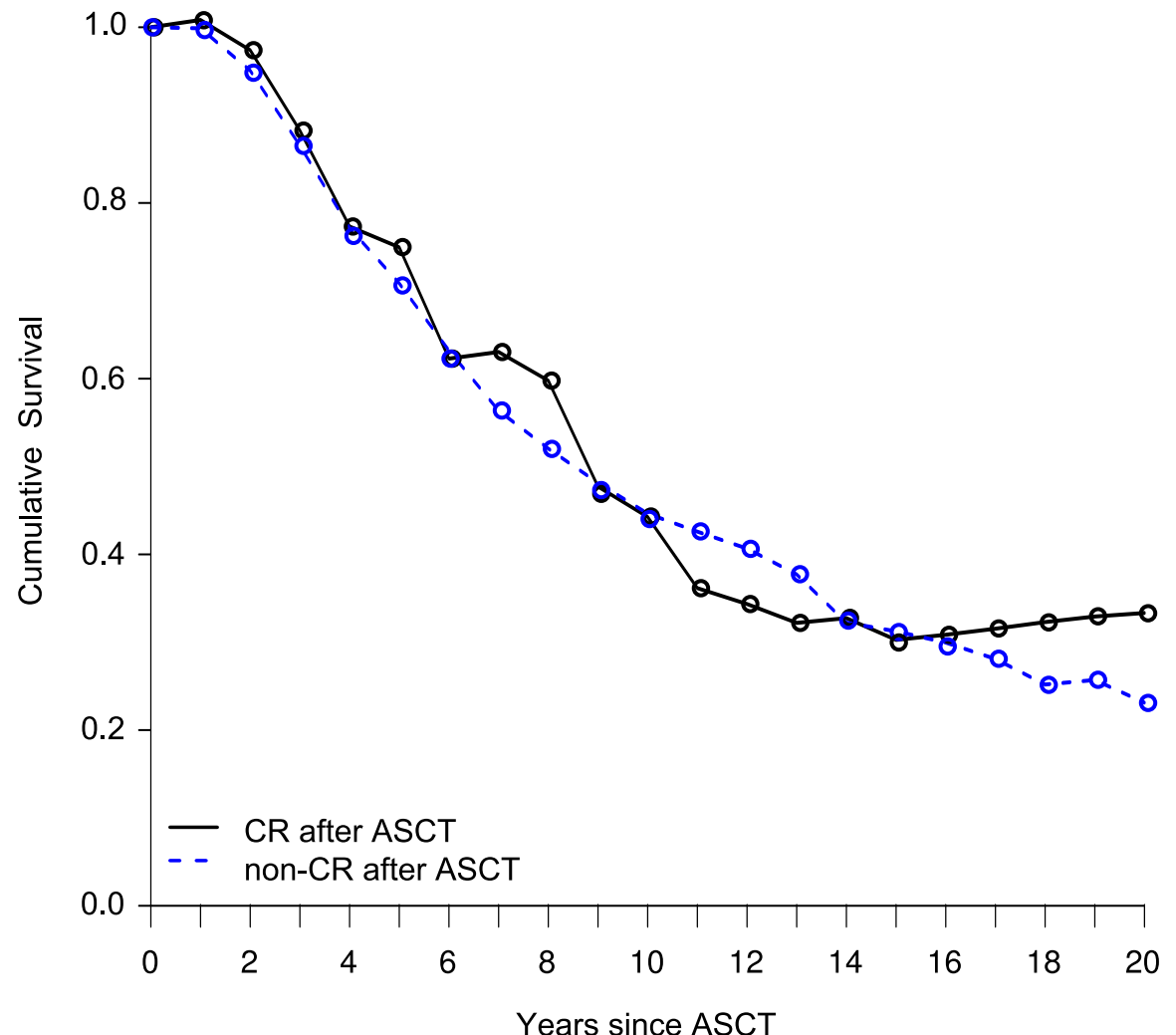

Supplement: Supplementary file 5 — Figure S5. Relative survival stratified by type of induction therapy (A) and response achieved after ASCT (B). [file CAM4-7-307-s005.pdf]
