# Supplementary material for: Analysis of long‐term survival in multiple myeloma after first‐line autologous stem cell transplantation: impact of clinical risk factors and sustained response
Source: Cancer Med. 2017 Dec 28;7(2):307–16. doi: 10.1002/cam4.1283 (PMC5806105; doi:10.1002/cam4.1283)
Supplement: Supplementary file 6 — Table S1. Details on induction regimens. Details on the most commonly applied induction regimens in our cohort are given.Table S2. Multivariate analysis of possible influence factors on PFS and OS – subgroup analysis of patients with novel agent‐based induction therapy.Table S3. Landmark analyses. Multivariate analysis of possible impact factors on OS is given at 1, 3, and 5 years after ASCT landmarks. [file CAM4-7-307-s006.docx]

#### Supplementary information

**Suppl. Figure S1: Progression-free survival (a) and overall survival (b) stratified by response achieved after ASCT.** EBMT response criteria are applied with CR – complete response, PR – partial response, MR – minimal response, and PD – progressive disease. Due to the very small number of patients with stable disease, data not shown.

**Suppl. Figure S2: Simon-Makuch plots of progression-free survival (a) and overall survival (b) stratified by type of maintenance therapy.** Simon-Makuch plots show PFS and OS according to no maintenance therapy, maintenance therapy with interferon α or with novel agents (i.e. thalidomide, bortezomib or lenalidomide). Maintenance therapy is assessed as a time-dependent variable thus accounting for an individual’s possible change from ‘no maintenance’ to ‘maintenance’ over time.

**Suppl. Figure S3: Landmark analysis at 1-year after ASCT.** Patients are stratified by sustained complete response (sustained CR), sustained inferior response (sustained non-CR), loss of complete response (lost CR) and loss of inferior response (lost non-CR).

**Suppl. Figure S4: 5-year (a) conditional survival for the entire patient cohort as well as 3-year conditional survival stratified by response achieved after ASCT (b).** EBMT response criteria are applied with CR – complete response, PR – partial response, MR – minimal response, SD – stable disease, and PD – progressive disease.

**Suppl. Figure S5: Relative survival stratified by type of induction therapy (a) and response achieved after ASCT (b).**

**Supplemental Table S1: Details on induction regimens.** Details on the most commonly applied induction regimens in our cohort are given.

**Supplemental Table S2: Multivariate analysis of possible influence factors on PFS and OS – subgroup analysis of patients with novel agent-based induction therapy.**

**Supplemental Table S3: Landmark analyses.** Multivariate analysis of possible impact factors on OS is given at 1, 3 and 5 years after ASCT landmarks.
